# The “Dental Digest” and Dental Colleges

**Published:** 1899-07

**Authors:** 


### THE "DENTAL DIGEST" AND DENTAL COLLEGES.

It is not surprising that the editor of the *Dental Digest* should labor through several pages of editorial, in the May number, to demonstrate the great value of examining boards and the inefficiency of the National Association of Dental Faculties, but it is astonishing that one so generally conversant with the work of the dental profession should deliberately, and apparently with malice aforethought, attempt to injure the work of the Faculties. Had he confined his criticism to simple opposition to that body, it would not have been considered of sufficient importance to notice, for the editor has, for some unexplained reason, always been in apparent opposition to colleges, as at present organized.

Truthful criticism is always valuable and welcomed, but when it descends to statements giving a perversion of facts, it becomes a serious matter. The following quotation illustrates this: "After great pressure had been brought to bear by *the earnest men of the profession for a higher standard,* the Faculties Association finally agreed to a requirement for better education before entrance, the same to take effect the following year; but at the next annual meeting the promised advance was refused, and the Association was left without any adequate standard of requirements, and no assurance was given the profession *that even this low standard would be enforced.* . . . It became evident to all intelligent practitioners that some other controlling power *must enforce* the reform." (Italics ours.)

Is this honest? The editor of the *Dental Digest* either did or did not know. If he were conversant with the history of the Faculties, he knew perfectly well the reasons for this so-called backward step. It has been repeatedly explained upon our pages, and reiteration will not be attempted here. If, on the other hand, he was ignorant of the final action of the Faculties, then why attempt an editorial that will be accepted as truth by all his readers. He was perfectly aware, or should have been, that whatever standard was adopted by the body would be enforced, and the colleges under its jurisdiction understood this fully. This Association will not move faster than it seems wise to go, whether the influence to move it proceeds from the *Dental Digest* or the National Association of Dental Examiners.

The editor forgot entirely to mention that the Faculties last year, at Omaha, adopted the following rule. It is quoted for his information.

"The minimum preliminary educational requirement of colleges of this Association, for the session of 1899–1900, shall be a certificate of entrance into the second year of a high school, or its equivalent, the preliminary examination to be placed in the hands of any State or county Superintendent of Public Instruction."

While this may not meet his views, it did that of the Association, and it was, unquestionably, as high a standard as it was wise to adopt at that time.

Had the editor of the *Dental Digest* been equally as wise, he would not have laid himself open to the suspicion that he quoted past history with the object of lowering the Faculties in the estimation of the dental profession.

It is not important, in our several relations, whether the editor does or does not support the National Association of Dental Examiners. If the *Dental Digest* finds its interest runs that way and not in the direction of the colleges, the latter have no cause to complain, but the editor will be wiser if he recalls the fact to memory that his warmest supporters in the Dental Protective Association have been these same educators, and to antagonize them in their efforts to raise the standard of dental education, slowly it may be, but steadily forward, by a suppression of facts, is not the course that will commend itself to right-thinking minds.

His article throughout exhibits an antagonistic animus, which, while it will be pleasant reading to a certain class, will be equally unpleasant to another and, perhaps, a far more important one, where the best interests of the dental profession are concerned.

